# Modelling interest in co-adoption of electric vehicles and solar photovoltaics in Australia to identify tailored policy needs

**DOI:** 10.1038/s41598-024-59318-7

**Published:** 2024-04-24

**Authors:** Elham Hajhashemi, Patricia Sauri Lavieri, Neema Nassir

**Affiliations:** Department of Infrastructure Engineering, The University of Melbourne, Melbourne, VIC Australia

**Keywords:** Sustainability, Photovoltaics, Climate-change mitigation

## Abstract

Electric vehicles (EVs) and solar photovoltaic systems (PVs) are two technologies that are gaining popularity in households as a means of reducing carbon emissions and improving energy security. However, little is known about the characteristics of households that adopt these technologies jointly. This study investigates the adoption patterns of electric vehicles and solar photovoltaics in Australia. We explain the likelihood of consumers belonging to four distinct groups (those who adopt both PVs and EVs, those who only adopt EVs, those who only adopt PVs, and those who adopt none) based on demographic and attitudinal factors. Using survey data from a representative sample of 2219 Australian heads of households, we found that dwelling ownership, ownership of a home energy management system, gender, and household size were significant predictors of the joint adoption of EVs and PVs. While both pro-environmental and pro-technology attitudes demonstrated a significant role in shaping PV-EV co-adoption patterns, the latter has a much stronger effect than the former. Based on the results, we identified that actions are needed in three key areas to encourage co-adoption: reducing technology adoption constraints associated with living arrangements (such as dwelling type and ownership), providing bundled financial incentives for both technologies, and fostering technology awareness and perceived usefulness among consumers.

## Introduction

In recent years, electric vehicles (EVs) have gained popularity as a technology to mitigate carbon emissions and enhance air quality. However, the extent to which they contribute to these objectives depends on the source of energy utilised for charging them. In the absence of a renewable energy source, switching to EVs would mostly displace emissions from the road to the power plant. Additionally, the increasing usage of EVs poses a challenge to the balance of electricity supply and demand. Research shows that EV owners tend to charge their vehicles during the evening, which coincides with the time when households already have the highest energy needs, leading to an upsurge in peak electricity consumption^1,2^.

To optimise the potential of EVs in reducing carbon emissions while minimising their negative impact on power systems, it is crucial to utilise more renewable energy sources for their charging. The adoption of solar photovoltaics (PVs) is gaining attention as a viable approach to meet this need^3,4^. Residential PVs incur installation costs but provide access to free and carbon–neutral electricity. Furthermore, the bi-directional charging capability of EVs can be leveraged to increase PV usage, reducing the stress on the grid and maximising self-consumption^5^.

Recent research in the United States shows that EV users who adopt PVs reduce their average hourly demand on the power grid by 1.1 kWh, demonstrating the potential for co-adoption of these technologies^6^. Additionally, studies have shown that home charging is the preferred location for EV users across the globe^1–3^, with prospective adopters exhibiting an even stronger preference for economic home charging options^4^. This underscores the relevance of promoting the joint adoption of PV and EV as a home-based and cost-effective charging option. Australia is already a global leader in residential PV installations per capita^7^, which creates a favourable environment to encourage PV-EV co-adoption. As such, to better shape strategies for transport decarbonisation, planners and policymakers must understand the market segments that are more and less prone to adopt these technologies.

Although the co-adoption of PVs and EVs can offer potential benefits, there has been limited research on consumer preferences and adoption patterns. Existing studies have primarily focused on PV-EV integration from a power systems and techno-economic analysis perspective, examining how this integration can alleviate pressure on the power grid, effectively balance electricity demand and supply derived from PVs, and lead to economic benefits such as savings on electricity bills^8–10^.

This study contributes to the PV-EV integration research field by investigating the characteristics of consumers that are likely to take part in one of four distinct adoption groups: those who adopt both PVs and EVs, those who only adopt EVs, those who only adopt PVs, and those who adopt none. By considering factors such as demographic variables, dwelling attributes, and attitudes toward technology and the environment, the study seeks to uncover major differences between these adoption groups. A hybrid discrete choice model^11^ that integrates endogenous continuous latent variables representing attitudinal constructs with the discrete outcome of group membership is estimated using data from a representative sample of Australian household heads. The results help us identify potential motivations, priorities, and barriers faced by different groups, providing substratum to inform tailored policy recommendations that can encourage a more sustainable transition toward fleet electrification.

The paper is structured as follows. The “Literature review” section provides an overview of the literature on the characteristics and motivations of individuals who have adopted PVs and EVs, exploring the potential linkage between these technologies. The “Method and data” section presents the research framework, data sources, and modelling methodology. The results of our analysis are reported in the “Results” section, which is followed by the “Policy implications”. Finally, section “Conclusion” highlights key study implications and suggests potential avenues for future research.

## Literature review

We provide a review of studies investigating the characteristics and motivations of individuals who have adopted EVs (Section “Characteristics and motivations of EV adopters”) and PVs (Section “Characteristics and motivations of PV adopters”). Then, we discuss the potential linkages between PV and EV adoption (Section “Linkage between PV and EV adoption”). Our review covers mostly studies that investigate the characteristics of PV and EV adopters separately because the current literature on co-adoption is scarce. The literature review is then used to position and justify the framework adopted in our study.

### Characteristics and motivations of EV adopters

Studies that characterise EV adopters typically utilise two primary types of predictors: sociodemographic characteristics at both individual and household levels, as well as attitudinal indicators. In terms of sociodemographic predictors, consistent trends have emerged across multiple studies, indicating a higher likelihood of EV ownership among males with higher levels of education and income. Additionally, EV adopters are typically employed full-time, approaching middle age, and tend to own multiple cars^12–17^. Furthermore, the presence of home charging infrastructure, often associated with owning a detached house, has been found to positively influence the adoption of EVs^12,16^. However, findings from the California and Norway markets (where EV penetration is passing the early adoption phase) suggest that although high-income families currently represent the largest group of adopters, current EV adopters have already started to exhibit characteristics similar to other car owners^13,18^.

When considering attitudinal indicators, technology enthusiasm and environmental concerns emerge as strong predictors of EV uptake^16,19,20^. Notably, while environmental concern is a significant predictor of EV adoption, technology enthusiasm stands out as the strongest predictor, with tech enthusiasts being nearly 40% more likely to adopt EVs^16^. Understanding these attitudinal indicators is crucial not only for predicting EV adoption but also for assessing the sustainable use of EVs. According to Peters et al.^21^, the motive behind adopting an EV plays a vital role in defining its sustainable usage as well as engaging in sustainable energy consumption. Adopting an EV for environmental reasons is often perceived as a pro-environmental statement that reinforces one's environmental identity and creates a positive feedback loop that propagates more sustainable choices.

### Characteristics and motivations of PV adopters

Similar to EV adoption, sociodemographic characteristics, including individual-level and household-level, along with attitudinal indicators, play a crucial role in characterising PV adopters. In terms of individual-level characteristics, the influence of age and education on PV adoption remains unclear, with varying findings in studies from different locations. Notably, research by Hansen et al.^22^ in Denmark and Sommerfeld et al.^23^ in Australia revealed that older individuals are more likely to adopt PVs. In contrast, Briguglio and Formosa^24^ conducted their study in Malta and found that younger households exhibit a higher propensity for PV adoption. Additionally, Hansen et al.^22^ observed that men with technical education backgrounds are more likely to be registered PV owners. However, Briguglio and Formosa^24^ and Sommerfeld et al.^23^ did not find higher education levels to be a significant influencing factor in their respective studies.

At the household level, dwelling features emerge as one of the most significant determinants influencing PV adoption. Ownership of detached dwellings facilitates the widespread adoption of PVs, while living in apartments and rental units presents a notable barrier to PV uptake due to the split incentive problem^22–27^. Split incentive refers to the governance challenges arising when the person or entity responsible for investing in and maintaining the solar energy system is different from the one using the generated solar energy, leading to disparities in the distribution of benefits and costs within an energy supply system^27,28^. In rental properties, landlords often own the roof space where solar panels could be installed, while tenants are the ones paying the electricity bills. This misalignment of incentives discourages landlords from investing in solar installations, as they may not directly benefit from reduced electricity costs. Consequently, this can lead to increased rent or dwelling management costs, which are not beneficial for renters^26,27^. Apartment residents face significant challenges in coordinating with other owners within the apartment complex to determine the equitable sharing of PV adoption benefits among different parties^26,27^. Furthermore, the limited roof space per household in apartments, compared to houses, poses an additional obstacle that hampers the feasibility of PV installations for apartment dwellers^26^.

High residential electricity consumption, which correlates with increased electricity bills, has also been identified as a crucial determinant for PV adoption in studies by Best et al.^26^ and Cohen et al.^29^. These studies reveal that factors such as the size of the house, number of household members, and ownership of electricity-intensive appliances serve as strong predictors of PV ownership.

While the importance of dwelling features in PV adoption is commonly agreed upon, the relationship between household income and PV adoption remains unclear and subject to varying findings across different regions. Several studies from Europe and Canada suggest a positive correlation between income and PV adoption^22,24,25^. However, research from Australia indicates that income may not play a significant role^23^. It appears that income itself might not be a decisive factor, but rather its importance lies in the ability to possess sufficient assets to cover the upfront costs associated with PV adoption. Best et al.^26^ findings from Australia confirm that income alone does not directly impact adoption rates. Instead, the overall net wealth of households, taking into account their assets and liabilities, emerges as a significant determinant. Furthermore, in Australia, Bondio et al.^30^ rated PVs as a technology primarily suitable for the middle class, implying that households considering PV systems would be both concerned about rising electricity bills and possess the financial capacity to meet the upfront expenses.

It is also important to note that the limited impact of income on PV adoption in Australia may be attributed to the presence of substantial subsidies. These subsidies, including rebates and feed-in tariffs, have been in place for over a decade and played a crucial role in driving PV uptake, contributing to one of the highest residential PV penetrations globally. For instance, Zander et al.^31^ identified that providing users with a 5 to 10-year security that they would be able to sell excess solar generation to retailers for a high tariff was a very influential factor in the choice to adopt PVs (only behind upfront installation costs). Moreover, a correlation was observed by Lan et al.^32^ between the adoption of PVs and changes in feed-in tariff regulations.

When it comes to attitudinal indicators, certain preferences and beliefs related to the environment can influence PV adoption. Environmental attitudes and beliefs, as well as behaviours such as participation in a green power scheme and practising energy conservation, indicate a higher likelihood of adopting PV systems^25,26^. However, Schelly^33^ findings from the United States revealed that environmental considerations alone are often not the primary driving force for PV adoption and homeowners may still choose to adopt rooftop PVs even if they do not have environmental concerns. Indeed, multiple studies found that financial gains and energy independence (reducing the reliance on the electrical grid) were the primary motivators for PV adoption^22,27,30^. In Australia, according to Zander^27^, installing PV systems for environmental reasons is more prevalent among females, well-educated individuals, and younger people.

In addition to pro-environmental attitudes, an interest in technology and the pleasure derived from the technical features of emerging energy systems were also identified as driving factors for PV adoption^33^. Some consumers also seem to seek PV adoption to feel as technology frontrunners, that is, they derive value from the status of being perceived as PV early adopters^22,34^.

### Linkage between PV and EV adoption

Results in the literature show a clear association between PV and EV adoption. For instance, Delmas et al.^35^ found a correlation between PV and EV ownership at an aggregate level, suggesting that regions with higher PV density also tend to have higher densities of EV ownership in California. They also identified a growth in this correlation over time. In a study conducted in Austria, Cohen et al.^29^ extended the investigation on the relationship between PV and EV adoption by considering one technology as an exogenous predictor of the other. The findings revealed a robust correlation, indicating a 31% higher likelihood of owning a PV if an EV is also owned, and a 7.1% higher likelihood of owning an EV if a PV is owned. To address concerns about endogeneity between PV and EV adoption decisions, they also employed a bivariate probit model and further analysed the impact of current PV ownership on future EV purchase. The results indicated that current PV owners were 21% more likely to intend to purchase an EV in the following five years compared to non-PV owners. Notably, PV ownership had the strongest impact on predicting EV adoption, surpassing factors like high income and a pro-environmental attitude.

Using a similar approach, Gezelius and Mortazavi^36^ examined household data from ten European countries to analyse a potential connection between PV and EV adoption while controlling for endogeneity between both decisions. Their findings showed a household's likelihood of owning an EV increased by 30% if it also had solar panels. Moreover, dwelling type, having a smart meter, and pro-environmental attitudes were found to be significant predictors of PV ownership, while owning an extra car and having pro-environmental attitudes were significant for EV ownership.

Gu et al.^37^ investigated the inverse relationship. That is, they looked into the impact of EV purchase intention on the desire to invest in PV and heat pump installations. Using data from a stated choice experiment in Austria, the study revealed that households with a preference for purchasing EVs showed a higher inclination to invest in home renewable energy equipment, particularly PVs. The likelihood of purchasing a PV increased by 6.99% when households opted for an EV over an internal combustion engine vehicle (ICEV).

Finally, a California study by Sharda et al.^38^ confirms the interconnectedness of PV and EV adoption. While a two-way relationship exists, owning an EV had a stronger influence on owning PVs. Beyond demographics, their research suggests that personality traits (extroversion) and social connections to existing EV/PV owners can also influence adoption decisions.

The studies described above developed models that uncover how much owning one technology positively influences the ownership of the other, suggesting that consumers perceive PVs and EVs as complementary products. However, the studies do not acknowledge the heterogeneity in consumer groups, in the sense that the co-adoption of technologies may be an obvious choice to one segment of people but not to others (e.g., some may consider only owning PVs or EVs). Consequently, they are unable to characterise consumer segments and identify potential strategies that can facilitate the co-adoption among those who are likely to adopt only one of the technologies or are not interested at all. The current study is designed to fill this gap, as our proposed model examines specific attitudinal and socio-demographic characteristics that differentiate consumers in their likelihood to exclusively adopt EVs, exclusively adopt PVs, co-adopt both technologies, and adopt neither. This approach produces rich results that can be used to guide adoption pathways through specific consumer-centred policies.

Moreover, while the studies considering the linkage between PV and EV adoption have considered the influence of environmental attitudes, they ignored that technology interest and savviness are also important predictors of the adoption of emerging technologies^16,19,33^. Therefore, in the current study, we examine the impact of both psychosocial constructs. Unlike the above studies, we consider that these constructs may not be directly observable, and we introduce them as endogenous stochastic latent variables in our model.

## Method and data

This section begins by presenting the Conceptual framework, which identifies the explanatory and latent variables that influence the adoption of PV and/or EV. Subsequently, the Modelling approach, Data, and Sample description are described.

### Conceptual framework

**Figure 1:**
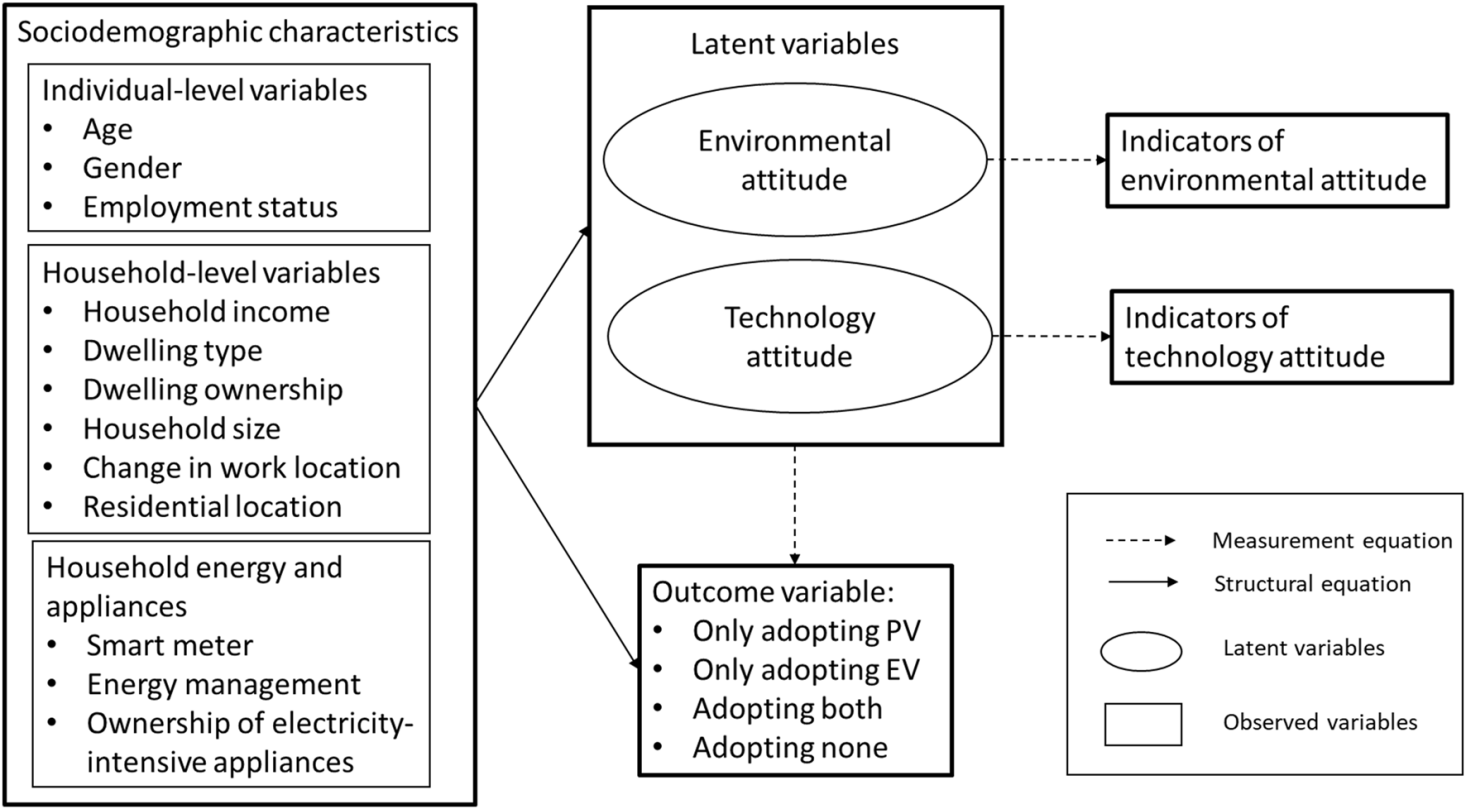
Conceptual framework.

In this paper, consumer choice to adopt PV and/or EV is explained using the exogenous explanatory variables and endogenous latent variables presented in the conceptual framework in Fig. 1. The choice is presented as a nominal dependent variable with four categories: (1) only adopting PV, (2) only adopting EV, (3) adopting both PV and EV, and (4) adopting none.

Based on the literature review, a set of socio-demographic and two latent variables representing the environmental attitude and technology attitude were used as determinants of the main outcome variable (PV or/and EV adoption). Sociodemographic characteristics are divided into individual-level (gender, age, and employment status), household-level (income, household size [continuous measure of the total number of individuals within each household], dwelling type, dwelling ownership, current change in work location, and residential location), and household energy and appliances (ownership of smart meter, energy management system, and electricity intensive appliances, such as a pool or multiple refrigerators) variables.

Constructs representing attitudes towards the environment and technology were extracted based on a factor analysis of eight attitudinal items elicited in the survey using 5-level Likert scales. The first latent variable, pro-environmental attitude, is used to capture how important climate change is to the consumer and their belief of having the power to change this issue by acting differently. This latent variable is relevant because it reflects the growing awareness and concern about environmental sustainability among individuals. As the effects of climate change become more apparent, consumers are increasingly recognising the need to adopt greener technologies and practices to mitigate its impact. Those with a stronger pro-environmental attitude are more likely to prioritise sustainable choices and seek out environmentally friendly alternatives. The second latent variable represents an individual's interest in technology, specifically related to the adoption of new technologies and use of smart energy management. This latent variable is relevant because both PV and EV are emerging technologies, and it is expected that individuals with a positive technology attitude are more likely to embrace new technologies and recognize their potential benefits, making them more inclined to adopt PVs and EVs. By incorporating these latent variables into the analysis, we can examine the influence of environmental and technology attitudes on PV and EV adoption. Gaining insights into the underlying psychosocial factors that drive consumer behaviour enables policymakers and other stakeholders to design targeted interventions and strategies that encourage the adoption of sustainable technologies.

### Modelling approach

Considering the nominal discrete nature of the outcome and the latent constructs, we utilise an integrated choice and latent variable (ICLV) modelling approach. This model incorporates the effects of latent variables on choice behaviour, allowing researchers to explore how unobserved factors, such as attitudes or preferences, influence individuals' decision-making processes. The ICLV model consists of two components: a discrete choice model and a latent variable model^11^. The components of the modelling framework are presented in Fig. 1. Next, we will present the mathematical formulation of the ICLV model^39,40^.

In our application, we have four distinct adoption groups and the discrete choice component of the model estimates the utility associated with belonging to each of these groups, taking into account the explanatory and latent variables. The utility derived by individual $n$ when belonging to group $i$ is described by Eq. (1).

1 $${U}_{n,i}= {V}_{n,i}+ {\epsilon }_{n,i}$$

Traditionally, in a discrete choice model, ${U}_{n,i}$ is the stochastic utility that is a function of the systematic utility $({V}_{n,i})$ and an error term ${(\epsilon }_{n,i})$ following a type I extreme value distribution. However, in an ICLV, ${V}_{n,i}$ is also stochastic because of the random effects incorporated via latent variables, as represented in Eqs. (2) and (3).

2 $$V_{n,i} = \delta_{n,i} + \beta_{i} x_{n,i} + \lambda_{i} \alpha_{n,i}$$

where ${\delta }_{n,i}$ is an alternative specific constant, ${x}_{n,i}$ is the vector of observed variables, for example, sociodemographic variables (in this study we only have individual related explanatory variables and no alternative specific variables). ${\alpha }_{n,i}$ refers to latent variables, and ${\beta }_{i}$ and ${\lambda }_{i}$ are vectors of estimated coefficients.

The latent variable component of the model contains structural and measurement models. The structural model part is shown in Eq. (3) and it estimates a vector of parameters (${\gamma }_{l})$ showing how sociodemographic variables ${(z}_{n})$ influence attitudes and preferences, which are represented by the latent variables (${\alpha }_{n,l}$), while ${\eta }_{n,l}$ is the associated random disturbance that follows a standard normal distribution across individuals ($l$ =1,…, L, where L is the total number of latent variables, in our case 2).

3 $$\alpha_{n,l} = \gamma_{l} z_{n} + \eta_{n,l}$$

The measurement model part uses an ordered logit model to construct the latent variables based on the responses to 5-level Likert scale attitudinal indicators ($s$). The measurement model is presented in Eq. (4).

4 $$L_{{I_{n,s} }} = \mathop \sum \limits_{p = 1}^{5} \delta_{{I_{n,s,p} }} \left( { \frac{{e^{{\tau_{{I_{s,p} }} - \zeta_{l,s} \alpha_{n,l} }} }}{{1 + e^{{\tau_{{I_{s,p} }} - \zeta_{l,s} \alpha_{n,l} }} }} - \frac{{e^{{\tau_{{I_{s,p - 1} }} - \zeta_{l,s} \alpha_{n,l} }} }}{{1 + e^{{\tau_{{I_{s,p - 1} }} - \zeta_{l,s} \alpha_{n,l} }} }}} \right)$$

${L}_{{I}_{n,s}}$ represents the likelihood of the observed value of an individual’s response ${I}_{n,s}$ to an indicator ($s$). ${\delta }_{{I}_{n,s,p}}$ takes the value of 1 if individual n selects response p (p: 1…,5) for indicator s. The parameter ${\tau }_{{I}_{s,p}}$ is estimated as the threshold value, where the normalisation condition for ${\tau }_{{I}_{s,0}}$ is set to -∞ and ${\tau }_{{I}_{s,5}}$ is set to + ∞ so that we estimate the four intermediate values. ${\zeta }_{l,s}$ estimates the impact of ${\alpha }_{l}$ on ${I}_{s}$. A significant estimate for ${\zeta }_{l,s}$ shows that the latent attitude ${\alpha }_{l}$ has a statistically significant impact on the answers provided to the attitudinal question ${I}_{s}$.

Equation 5 represents the equation that calculates the likelihood of individual n belonging to group $i$, conditional on ${\alpha }_{n}$ and $\beta$.

5 $$L_{{C_{n} }} \left( {\beta ,\alpha_{n} } \right) = \frac{{e^{{V_{n,i} }} }}{{\mathop \sum \nolimits_{j = 1}^{4} e^{{V_{n,j} }} }}$$

The joint log-likelihood of all model components is given in Eq. (6). Both of the model components, the component relating to the choice and the component related to attitudinal questions are a function of latent variable. This is why to jointly estimate these models, the entire likelihood function is integrated over the random component in the latent variable (${\eta }_{n}$). The Apollo R-programming package is utilized for estimating the ICLV model^41^.

6 $${\text{LL}}=\sum_{n=1}^{N}{\text{log}}\int {L}_{{C}_{n}}(\beta ,{\alpha }_{n})\prod_{s=1}^{8}{L}_{{I}_{n,s}} \Phi ({\eta }_{n})d{\eta }_{n}$$

To mitigate the potential for reaching local maxima during estimation, we utilised a search strategy recommended in the Apollo package^41^. This involved employing multiple starting points to systematically eliminate unlikely solutions and enhance the probability of discovering the optimal one.

### Data

The data used for this analysis is open source and were collected through a web-based survey conducted by Essential Research for Energy Consumers Australia in 2022. The target group for the survey consisted of individuals who are heads of households, aged 18 and above, and actively involved in decision-making regarding electricity and gas matters within their households across Australia. This survey is conducted annually and explores the attitudes and activity of residential energy consumers by asking questions about how they use power and energy technologies, their attitude to new technology, and their view on the future of energy. For further information, please refer to the details provided by Energy Consumers Australia on their website^42^. In this section, we highlight the survey aspects pertinent to the present analysis, as listed in section "Method and data".

The choice variable is not the outcome of a direct question in the survey and is defined based on respondents' PV and EV adoption status. PV and EV adoption were elicited separately using four possible options: (1) currently own, (2) intending to purchase in the next 12 months, (3) considering, but not intending to purchase in the next 12 months, and (4) not intending to purchase. To define the outcome variable of our model, respondents who owned, intended, and considered buying PV and EV were categorised as adopting both PV and EV, respondents who owned, intended, or considered buying EV but did not intend to purchase PV were categorised as only adopting EV. Analogously, respondents who owned, intended, or considered buying PV but did not intend to purchase an EV were categorised as only adopting PV. Lastly, respondents who did not intend to buy an PV nor an EV were categorised as adopting none. The decision to merge current adopters/owners with those intending and considering adoption was driven by the aim of obtaining a comprehensive understanding of the adoption landscape and the factors influencing adoption decisions across different stages. By solely focusing on current owners, we would miss out on understanding the needs and motivations of individuals who are considering and intending to adopt PV and/or EV technologies (and we would have a myopic perspective of early adopters only). By including them in the same category as current owners, we can develop more effective policies that consider the necessities of prospective adopters, potentially accelerating the overall adoption process. Additionally, merging these categories increases the sample size of the different groups, resulting in improved statistical power and analysis reliability (helping to mitigate biases that may arise from small sample sizes). This is particularly crucial in Australia, where the current number of EV owners is relatively low.

### Sample description

Table 1 presents the descriptive statistics of dependent and explanatory variables for the final clean sample, consisting of 2219 respondents. Most respondents are still not willing to adopt PVs or EVs, with the smallest group being those who are willing to adopt only EVs and not PVs. This pattern is expected in Australia, where EV adoption is lagging but PV adoption rates are relatively high (33%, according to the Australian PV Institute^7^).

**Table 1:** Descriptive statistics of dependent and explanatory variables.

Variable	Count	Sample %	Census %
Outcome variable (choice)			
Only adopting PV	667	30.1	–
Only adopting EV	173	7.8	–
Adopting both	527	23.7	–
Adopting none	852	38.4	–
Individual-level sociodemographic variables			
Gender			
Female	1197	53.9	51.2
Male	1022	46.1	48.8
Age			
18–34	484	21.8	29.4
35–54	712	32.1	33.9
55–74	790	35.6	26.9
75 or more	233	10.5	9.8
Employment status			
Full-time	742	33.4	–
Part-time	277	12.5	–
Retired	743	33.5	–
Other	457	20.6	–
Household-level sociodemographic variables			
Income			
Missing	151	6.8	–
40,000 or less	563	25.4	19.8
40,001 to 80,000	620	28.0	25.0
80,001 to 120,000	438	19.7	18.7
120,001 or more	447	20.1	36.5
Household size	Mean: 2.5		–
Dwelling type			
Detached house or townhouse	1809	81.5	70.0
Apartment and flat	410	18.5	30.0
Dwelling ownership			
Own	1478	67.0	66.0
Rent	732	33.0	34.0
My household works from home/studies from home more in the last 12 months			
Yes	539	24.3	–
No	1680	75.7	–
Residential location			
Inner metro	739	33.3	–
Outer metro	647	29.2	–
Provincial	270	12.2	–
Rural	563	25.3	–
Household energy and appliances variables			
Have a smart meter in our household			
Yes	904	40.7	–
No	1315	59.3	–
Have a home energy management system			
Yes	214	9.6	–
No	2005	90.4	–
Have a swimming pool or spa pool			
Yes	261	11.8	–
No	1958	88.2	–
Have three or more fridges/freezers			
Yes	499	22.5	–
No	1720	77.5	–

According to the data providers, the sample is considered representative of the population of household heads in terms of age and gender. However, it is worth noting that we do not have access to the specific distribution of this population from Census data. Therefore, we compare the distribution of selected sociodemographic characteristics in the sample with that of the Australian driving age population^43,44^. The sample shows a slightly higher share of women and a lower share of young adults (between 18 and 34 years old) compared to the driving age population. This is an expected difference considering that the survey targeted individuals who make electricity-related decisions in the household. While the share of home owners and renters is equivalent to the population, we observe an underrepresentation of multi-unit building dwellers^45^. This limitation is particularly relevant to the problem investigated in this study because both PV installation and EV charging face more significant barriers in apartment complexes. An aggregate analysis based on this sample would thus be biased, but by using a disaggregate model that can capture both observed and unobserved individual heterogeneity we expect to extract important insights even for this underrepresented group.

Regarding household energy and appliances related variables, smart meter ownership rates are much higher than home energy management system ownership rates, with 40.7% of respondents having a smart meter and only 9.6% having an energy management system. The discrepancy in ownership rates between smart meters and home energy management systems in Australia can be largely attributed to the extensive rollout of smart meters facilitated by government initiatives and utility companies, especially in Victoria^46^. Additionally, the perceived benefits of smart meters, such as accurate and real-time energy usage information, maybe more widely recognized and valued by consumers compared to the advanced features offered by home energy management systems. A relatively low percentage of households own electricity-intensive appliances, with 11.8% owning swimming pools or spa pools and 22.5% having three or more refrigerators.

Table 2 displays the attitudinal indicators and their response distributions, which were measured on a five-point Likert scale. Respondents were asked to choose from five levels of agreement, from strongly disagree to strongly agree. For analysis purposes, we assigned a value of 5 to the highest level of favourable attitudes toward the environment and technology, and a value of 1 to the least favourable level. The last four indicators of environmental attitudes had their scale inverted to ensure that they were all capturing the underlying construct monotonically. Respondents generally express a desire for action on climate change issues. Regarding technology attitudes, only 6% consider themselves early adopters, while around 20% are strongly inclined to use technology to manage bills and learn about new ways of generating, storing, and distributing electricity.

**Table 2:** Frequency of response to ten attitudinal indicators.

Environmental attitude					
	Strongly disagree 1	Somewhat disagree 2	Neither agree nor disagree 3	Somewhat agree 4	Strongly agree 5
Climate change is something we need to act on now (Env1)	6.2%	4.6%	14.5%	24.8%	49.9%

	Strongly agree 1	Somewhat agree 2	Neither agree nor disagree 3	Somewhat disagree 4	Strongly disagree 5
I agree that climate change is occurring, however, it is too late to do anything about it (Env2)	5.2%	12.1%	24.6%	32.9%	25.2%
Fluctuations in the climate are all part of the natural cycle (Env3)	19.3%	31.3%	23.4%	15.4%	10.5%
Whatever Australia does to address climate change won’t make a difference anyway (Env4)	14.2%	17.2%	19.7%	26.5%	22.4%
Other issues are more important than climate change (Env5)	16.4%	20.5%	28.3%	19.9%	14.9%

Technology attitude					
	Strongly disagree 1	Somewhat disagree 2	Neither agree nor disagree 3	Somewhat agree 4	Strongly agree 5
I consider myself something of an early adopter of new technologies (Tech1)	18.1%	26.3%	25.5%	23.7%	6.4%
I am interested in new technology to help manage my household energy bills (Tech2)	5.4%	7.9%	23.2%	42.9%	20.6%
I am interested in learning about new ways of generating, storing, and disturbing electricity (Tech3)	5.2%	9.2%	23.2%	41.3%	21.1%

### Factor analysis

We conducted an exploratory factor analysis using Principal Axis Factoring to examine the relationships among the indicators and identify latent variables. Two factors emerged, both demonstrating strong consistency, as all loadings surpassed the threshold of 0.4. We labelled the first factor 'Pro-Environmental Attitude,' as it comprised all five indicators related to environmental attitudes. The second factor, which included all three indicators associated with technology attitudes, was named 'Technology Interest.' The outcomes of the factor analysis, including the two resulting factors and the rotated factor loadings after Varimax rotation, are presented in Table 3. It is important to note that the factor analysis coefficients were not utilized in the model; the factor analysis was solely conducted to identify the optimal indicators for the latent constructs.

**Table 3:** Factor analysis on two latent variables.

Indicator code	Attitudinal question	Pro-environment attitude	Technology interest
Env1	Climate change is something we need to act on now	0.633	
Env2*	I agree that climate change is occurring, however, it is too late to do anything about it	0.413	
Env3*	Fluctuations in the climate are all part of the natural cycle	0.660	
Env4*	Whatever Australia does to address climate change won’t make a difference anyway	0.820	
Env5*	Other issues are more important than climate change	0.794	
Tech1	I consider myself something of an early adopter of new technologies		0.571
Tech2	I am interested in new technology to help manage my household energy bills		0.777
Tech3	I am interested in learning about new ways of generating, storing, and disturbing electricity		0.720

* The original measurement of indicators Env2-Env5 reflected a lack of pro-environmental attitude. In line with our analysis focusing on pro-environmental tendencies, we inverted the scales of these indicators for consistency. Hence, their factor loadings appear positive in the table.

## Results

The following sections present the results of the ICLV model components including the Latent variable structural model, the Latent variable measurement model, and the Discrete choice model. In section "Average treatment effects", we calculate the average treatment effect of each statistically significant explanatory coefficient and compare their magnitudes to identify the most significant determinants of PV and EV co-adoption and separate adoption.

### Latent variable structural model

Table 4 displays the structural associations between socio-demographic variables and the latent constructs (also note that these socio-demographic variables have a direct impact on the nominal outcome that will be discussed in section "Discrete choice model"). Gender has a significant effect on attitudes towards the environment and technology. Men tend to display lower levels of pro-environmental attitudes, but higher levels of tech interest. These findings align with existing literature that highlights women's tendency to hold stronger pro-environmental attitudes^47^. This inclination can be attributed to women's greater inclination towards prosocial values compared to men^48,49^. As a result, women are more likely to engage in environmentally conscious behaviours^50^. Furthermore, studies also support the notion that, in general, men display a greater interest in technology compared to women, particularly they tend to have stronger beliefs of the societal benefits of technology and self-confidence in acquiring and effectively utilising technological gadgets^51,52^.

**Table 4:** Estimation results for structural equation model for latent variables.

Latent variable	Pro-environmental attitude		Technology interest	
	Est	t-stat	Est	t-stat
Gender (female)				
Male	− 0.206	− 4.38	0.312	5.86
Age (75+)				
18–34	0.361	3.96	0.814	10.23
35–54	0.269	3.10	0.341	4.91
55–74	0.160	2.00	–	–
Full-time worker	–	–	0.264	3.89
Income (40,000 or less)				
40,001–80,000	–	–	0.181	2.77
80,001–120,000	–	–	0.248	3.16
120,001 or more	0.237	3.91	0.279	3.22
Household size	− 0.092	− 4.23	0.059	2.33
Correlation between latent variables				
Pro-environmental attitude	1.000	n/a		
Technology interest	0.265	9.17	1.000	n/a

(–) indicates that this variable was not significant and was excluded from the model specification.

(n/a) indicates not applicable.

Younger adults generally show a greater level of enthusiasm for both the environment and technology compared to their older peers. Research consistently suggests that younger individuals display greater environmental concern than older individuals^53,54^. Furthermore, it is widely recognized that younger individuals possess advanced technology literacy, attributed to their early exposure to information and communication technology (ICT)^55,56^. This heightened technological proficiency not only increases their interest in technology but also renders technology-based interventions more suitable for them^57^.

Full-time workers tend to exhibit a higher level of tech interest, which can be attributed to their regular exposure to technology in their professional lives^56^. This exposure fosters greater comfort, familiarity, and expertise, resulting in an increased interest in technology. Individuals from higher-income households are more likely to hold pro-environmental attitudes, as research consistently shows a positive relationship between income and pro-environmental attitudes^58^. This connection may be attributed to the tendency of individuals with higher income levels to adopt post-materialistic views that prioritise quality of life and environmental sustainability^59^. Additionally, a higher income level is associated with a greater level of tech interest, as evidenced by other studies^60,61^. This association can be explained by the increased purchasing power of higher income earners, which grants them early access to new technologies. Finally, families with larger household sizes are less likely to hold pro-environmental attitudes but display a higher level of tech interest. This correlation aligns with previous findings suggesting that households with children (typical of larger families) often prefer suburban residential settings, spacious homes, and a lifestyle reliant on automobiles, which may lead to a less environmentally friendly lifestyle^61,62^. However, the focus on technology in these households may arise from the perceived benefits it offers in managing and facilitating the complexities of a larger household. Moreover, there may be a greater potential for shared benefits that justify investments in technology. For example, a smart home system could be considered more justifiable for a family of five compared to a single individual.

The correlation between latent variables is shown at the bottom of Table 4. Technology interest is positively correlated with pro-environmental attitudes. This finding aligns with previous research^63,64^ and is intuitively logical. Individuals with a strong interest in technology are likely to be more receptive to innovative solutions, potentially including those that promote sustainable living. This openness could manifest in a greater willingness to adopt efficient technologies like renewable energy systems or smart home devices designed to minimize energy consumption.

### Latent variable measurement model

Table 5 presents the results of the measurement relationship of the estimated model. All indicators demonstrated statistically significant associations with the latent attitudes at a 99% confidence level. Moreover, the direction of these associations aligned with the anticipated expectations. The positive estimation value ($\zeta$) for all indicators means that a higher value for latent variables leads to a higher level of agreement with the indicators.

**Table 5:** Estimation results for measurement model for latent attitudes.

Latent variable	Indicator	Estimate (${\varvec{\zeta}})$		Threshold 1 (${{\varvec{\tau}}}_{1}$)		Threshold 2 (${{\varvec{\tau}}}_{2})$		Threshold 3 (${{\varvec{\tau}}}_{3}$)		Threshold 4 (${{\varvec{\tau}}}_{4}$)	
		Est	t-stat	Est	t-stat	Est	t-stat	Est	t-stat	Est	t-stat
Pro-environmental attitude	Env1	2.345	22.51	− 4.723	− 18.11	− 3.752	− 15.54	− 2.032	− 9.21	− 0.112	− 0.53
	Env2	0.924	17.27	− 3.269	− 25.31	− 1.859	− 18.32	− 0.438	− 4.74	1.246	12.90
	Env3	1.835	24.09	− 2.259	− 12.76	− 0.052	− 0.31	1.455	8.51	3.031	16.34
	Env4	2.731	22.19	− 3.644	− 13.46	− 1.705	− 6.82	− 0.052	− 0.21	2.321	9.06
	Env5	2.687	22.36	− 3.290	− 12.45	− 1.146	− 4.70	1.122	4.62	3.199	12.34
Technology interest	Tech1	1.350	21.24	− 0.980	− 8.82	0.802	7.03	2.289	17.61	4.652	26.60
	Tech2	2.420	17.40	− 3.305	− 14.81	− 1.650	− 8.87	0.883	4.85	4.676	17.47
	Tech3	1.915	19.89	− 3.026	− 16.84	− 1.372	− 9.22	0.717	4.92	3.822	19.90

### Discrete choice model

The results of the choice model are presented in Table 6. The coefficients represent the direct effects of variables on the utilities of adopting only PV, only EV, and both PV and EV, with the base alternative being adopting none. Several nesting structures were explored to address the correlation between alternatives, including all potential nesting configurations among the four alternatives (only adopting PV, only adopting EV, adopting both PV and EV, and adopting none). However, none were found to be statistically significant. This indicates that all correlations between alternatives are effectively captured through latent variables in the model.

**Table 6:** Estimation results for choice model components.

Variables	Base: adopting none					
	Only adopt PV		Only adopt EV		Adopt PV and EV	
Latent variables	Estimate	t-rat	Estimate	t-rat	Estimate	t-rat
Environment attitude	–	–	0.297	2.95	0.375	5.42
Technology attitude	0.509	7.29	0.780	6.96	1.125	12.30
Individual-level sociodemographic variables						
Gender						
Male	–	–	0.389	2.25	0.574	4.70
Employment status						
Retired	0.484	4.09	–	–	–	–
Household-level sociodemographic variables						
Income						
120,000 or more	–	–	0.497	2.57	–	–
Dwelling type						
Detached house or townhouse	0.968	5.55	–	–	0.787	4.11
Dwelling ownership						
Own	1.660	11.99	–	–	1.472	9.55
HH size	0.135	2.49	–	–	0.213	3.71
My household works from home/studies from home more in last 12 months						
Yes	–	–	0.448	2.48	–	–
Residential location						
Inner metro	–	–	0.285	1.67	–	–
Have smart meter in our household	–	–	–	–	0.281	2.33
Have a home energy management system	0.933	4.01	–	–	1.499	6.44
Have swimming pool or spa pool	0.538	3.04	–	–	0.452	2.22
Have three or more fridges/freezers	0.348	3.00	–	–	–	–
Alternative specific constant	− 3.195	− 13.88	− 2.752	− 14.49	− 4.294	− 15.98
Goodness of fit measures						
Adj.Rho-squared vs equal shares	0.1925					
Adj.Rho-squared vs observed shares	0.1193					
LL (start)	− 31,213.08					
LL (final, whole model)	− 25,472.70					
AIC	51,107.40					
BIC	51,569.49					
Number of observations	2219					

(–) indicates that this variable was not significant and has been excluded from the model.

In terms of the impact of latent variables, the results suggest that individuals with a pro-environmental attitude are more likely to adopt both PV and EV, as well as only EV. Interestingly, a pro-environmental attitude does not appear to play a significant role in the adoption of only PV, contradicting previous studies emphasising its influence on PV adoption^25,26^. It appears that financial gains take precedence over environmental motivations for PV adoption in Australia^27,30^. The prominence of economic motivations in PV adoption in Australia can be attributed to subsidies outlined in section "Characteristics and motivations of PV adopters".

Our findings confirm the anticipated technology interest effect. Individuals with higher levels of tech interest are more likely to adopt both PV and EV, as well as choose either only EV or only PV, compared to those who do not adopt any of these options. This aligns with previous studies by Brückmann et al.^16^ and Lane et al.^19^, which identified tech enthusiasm as a strong predictor of EV adoption, and Schelly^33^ who raised that the enjoyment derived from the technical aspects of energy systems are common motivating factors for PV adoption.

In addition to the indirect sociodemographic effects through the latent variables, results show direct sociodemographic effects on PV and/or EV adoption. Being male increases the probability of adopting only EV and both PV and EV, beyond the positive effect of being male through tech enthusiasm (and while males have lower pro-environmental attitudes, this indirect negative effect gets swamped by the magnitude of the positive direct effect of being male). These findings align with previous studies that have shown a higher tendency for males to adopt EVs^14,15,17,65^.

Retired individuals are more likely to adopt only PV, as supported by various reasons found in the literature. Retirement marks a significant economic life event, leading to decisions about housing, investments, and managing living on a fixed income^33^. They tend to perceive more value from ongoing benefits over the initial cost of PV installation, especially given concerns about uncertain future electricity prices, reduced retirement income, or dependence on pensions^23,30,66,67^. Moreover, retirees may be eligible for additional incentives, such as reduced upfront costs for individuals holding pensioner concession cards in specific regions like the Australian Capital Territory^68^.

A higher household income increases the probability of adopting only EV, in addition to the positive effects of income through environment and technology attitude. The absence of a significant direct income effect on PV adoption supports our earlier observation that subsidies in Australia are being effective in overcoming the upfront purchase cost barrier associated with this technology. On the other hand, the minimal subsidies for EV adoption do not have the same effect and these vehicles are still only accessible to affluent consumers^69^.

Our results also indicate that living in a detached house or townhouse, owning a house, and having a larger household size can have a positive effect on the adoption of PV and both PV and EV together. This outcome aligns with expectations, considering that living in apartments and rental units has been identified as a significant constraint for PV adoption^26^, as discussed in section "Characteristics and motivations of PV adopters". This result reinforces the importance of developing inclusive policies and strategies that target these consumer segments that are limited in their capability to install PVs as this may result in them being inequitably burdened with higher EV charging costs.

Living in a detached house or townhouse and owning a house do not seem to affect exclusive EV adoption, despite home charging usually being the preferred location for EV charging according to previous studies^1^. This result indicates that consumers in this group either do not perceive residential charging as a barrier or think that they can meet their travel needs by using public charging infrastructure. Indeed, exclusive EV adopters tend to work from home more frequently and live in metropolitan areas, which align with this situation. With lower mobility needs due to residing in inner metro areas and increased remote work, their charging requirements are reduced, enabling them to meet their charging needs through public and fast chargers.

In terms of household energy and appliances, individuals who utilise smart meters and home energy management systems to monitor and control their electricity consumption demonstrate a higher likelihood of adopting both PV and EV systems. This is expected, as having both PV and EV may require a more deliberate management of energy usage. Additionally, the presence of a home energy management system increases the probability of exclusively adopting PV. Home energy management systems empower consumers to actively control and optimise their home energy usage, leading to more efficient consumption patterns^70^. Consequently, individuals with energy management systems tend to be more mindful of their energy consumption and have a stronger inclination to enhance energy efficiency. The integration of PV systems with energy management further amplifies the promotion of efficient consumption behaviours. This is particularly significant since the pursuit of self-sufficiency and energy independence has been identified as a key motivating factor for PV adoption^22^.

Finally, as expected, households that own electricity-intensive appliances, such as swimming pools and three or more refrigerators, are more likely to only adopt PV. This is probably because they can increase the self-consumption of produced solar energy^29^. In addition to increasing the probability of adopting only PV, having a swimming pool also increases the probability of adopting both PV and EV, while having three or more refrigerators has an insignificant but positive effect on adopting both technologies. The negative alternative specific constant for all three options indicates that people still show resistance in adopting any of these options.

### Average treatment effects

To compare the magnitude of effects and identify the most significant determinants of PV and/or EV adoption, we employed the method outlined by Lavieri and Bhat^71^ to calculate the average treatment effects (ATEs) of explanatory variables. This includes the explicit consideration of latent variables as determinant variables, rather than translating their effects into corresponding demographic variables through the structural equation model results. This allows us to calculate ATEs for both direct demographic effects and the effects of the latent variables (instead of the overall demographic effects, which would be obtained by the summation of the direct and indirect socio-demographic effects) to create insights for policy formulation in Australia, leveraging the representative sample of household heads as discussed in section "Sample description".

#### Calculation

The ATE consists of the difference in probability of belonging to one group of adopters for a randomly selected individual when they are in a specific category $i$ of an explanatory variable versus another category $k$ ≠ $i$ (for example, being male versus female). We used this approach to estimate the ATE for each explanatory variable, following the process outlined by Lavieri and Bhat^71^.

$$\widehat{{ATE}_{ikj}}=\frac{1}{N} \sum_{n=1}^{N}([P\left({y}_{n}=j\right|{a}_{ni}=1)-P ({y}_{n}=j|{a}_{nk}=1)])$$

where ${y}_{n}$ is the nominal variable representing adoption group, and $j$ presents a specific adoption group that an individual belongs to (for example adopting only PV). ${a}_{ni}$ is the dummy variable for the category $i$ of the explanatory variable for the individual $n$ (N = 2219). $\widehat{{ATE}_{ikj}}$ represents the expected value change in the nominal category *j* of PV or/and EV adoption because of a change from category $k$ to $i$ of the explanatory variable. To calculate the effect, we begin by assigning the base category value to everyone in our sample. This involves setting the explanatory variable of interest to a value of ${a}_{nk}$ = 1, and computing the $P ({y}_{n}=j|{a}_{nk}=1)$. Next, we change the value of the variable to ${a}_{ni}$ = 1, and compute the probability of $P ({y}_{n}=j|{a}_{ni}=1)$.

We compute the ATE measure for the nominal categories of “only adopt PV”, “only adopt EV”, and “adopt PV and EV”, and for one combination of $i$ and $k$. For instance, for gender, the base category is “female”, while the changed category is “male”. Similarly, for employment status, the base category is “not-retired”, and the change category is “retired”. The results of ATE calculation are presented in Table 7. The mean and standard errors are obtained by utilizing 200 different draws from the sampling distributions of the estimated parameters and computing the fitted probabilities. Since latent variables are continuous, we examine what would happen if individuals transitioned from the lowest spectrum of pro-environmental attitude and technology interest to the highest spectrum.

**Table 7:** Average treatment effect.

Variable	Categories compared (base versus changed)	Only adopt PV		Only adopt EV		Adopt PV and EV	
		Est	St. err	Est	St. err	Est	St. err
Environment attitude	Min versus Max	0.000		0.015	0.0008	0.056	0.0011
Technology attitude	Min versus Max	0.009	0.0014	0.043	0.0009	0.217	0.0018
Gender	Female versus Male	0.000		0.015	0.0008	0.074	0.0012
Employment status	Not retired versus Retired	0.090	0.0017	0.000		0.000	
Income	Less than $120,000 versus $120,000 or more	0.000		0.038	0.0013	0.000	
Dwelling type	Not detached house or townhouse versus Detached house or townhouse	0.119	0.0018	0.000		0.048	0.0017
Dwelling ownership	Rent versus Own	0.213	0.0014	0.000		0.107	0.0012
HH size	1 versus 5	0.043	0.0021	0.000		0.082	0.0018
My household works from home more in last 12 months	No versus Yes	0.000		0.033	0.0011	0.000	
Residential location	Not inner metro versus Inner metro	0.000		0.018	0.0009	0.000	
Have smart meter	No versus Yes	0.000		0.000		0.040	0.0012
Have a home energy management system	No versus Yes	0.037	0.0024	0.000		0.163	0.0025
Have swimming pool or spa pool	No versus Yes	0.065	0.0019	0.000		0.024	0.0019
Have three or more fridges/freezers	No versus Yes	0.064	0.0017	0.000		0.000	

#### Results

The strongest factor influencing the exclusive adoption of PV systems is household dwelling ownership status, with household dwelling type being the second most influential determinant. This can be attributed to the fact that living in rental units and apartments are currently significant barriers to PV uptake^26^. For individuals who solely intend to adopt EVs, the strongest determining factor is technology interest. However, income is almost equally strong. This is an important distinction between the group that is just interested in EVs and the group that intends to co-adopt, as discussed earlier. That is, those who are planning to adopt EVs only are likely less sensitive to charging costs than the average consumer.

For those co-adopting PV-EV technology, the strongest determinants are technology interest, followed by the presence of a home energy management system and dwelling ownership. Suggesting that co-adoption requires both individuals to have the dwelling capability to install PVs and the interest in technology and energy management.

While technology interest is the primary predictor for both exclusive EV adoption and joint adoption of PV and EV, its impact is considerably greater for the latter. Pro-environmental attitude has a significantly stronger effect on joint adoption as well. However, the influence of technology interest is approximately four times greater than the environmental effect, underscoring the urgent need for consumers to become more familiar with emerging technologies.

The ATEs also show that other predictors, such as gender and current use of energy management systems, have a stronger effect on PV-EV co-adoption than on the exclusive adoption of one of the technologies. In this sense, we identify the importance of tailored policies to engage females in the integrated adoption of such technologies and campaigns to raise awareness of the potential benefits of home energy management.

## Policy implications

The joint adoption of EVs and PVs can facilitate the decarbonisation of motorised travel and reduce the potential stress that EV adoption growth may impose on the grid. In this section, we discuss the implications of the study results on the development of tailored policy recommendations that encourage PV-EV co-adoption. Based on the findings, we identified three main categories of policy actions: (1) reducing barriers to PV-EV adoption associated with living arrangements, (2) providing financial incentives, and (3) increasing technology awareness and interest.

### Reducing living arrangements’ barriers

This study indicates that dwelling ownership and type are the strongest predictors of interest in PV adoption in Australia. As expected, these variables (especially ownership) also significantly influence the intention to co-adopt PVs and EVs. However, dwelling type and ownership do not play a significant role in the adoption of EVs alone, suggesting that limitations associated with rental units and multiunit buildings may contribute to this group's exclusive adoption of EVs. Therefore, it is necessary to implement policies that enable all households, regardless of their living situation, to access PV technology or other cheap renewable resource for EV charging.

Specifically, policies are needed to facilitate PV installation in rental units and apartments. To facilitate PV adoption in rental dwellings, one solution is to introduce solar rebates for rental properties, similar to the program in Victoria, Australia. This program allows landlords to apply for a rebate before installation, with renters benefiting from lower electricity bills^72^. Additionally, shared solar presents a potential solution for apartment residents, enabling them to purchase or lease part of a larger PV system. To facilitate shared solar, new policies and business models, such as third-party-owned photovoltaic systems or building-integrated photovoltaics, must be developed and regulated^27,73^. Collective self-consumption (CSC) initiatives provide an alternative solution to reduce reliance on specific living situations for PV adoption. In CSC projects, end-users in space-constrained settings, like multi-tenancy buildings, collectively own energy generation and storage systems for self-consumption. By sharing costs and resources, CSC consumers overcome barriers to individual PV ownership, enabling residents who cannot install solar panels individually to benefit from solar energy. Implementing supportive policies and regulations can facilitate the development of CSC projects, ensuring equitable access to solar energy for all households^74^.

Enabling PV installations and shared use can solve part of the problem, but it is also necessary to ensure that renters and residents of multiunit buildings have adequate infrastructure to park and charge their vehicles. Options for affordable home charging installations and "right-to-install" legislation, granting tenants the authority to install charging stations without the need for building owners' approval, are important steps to achieve this goal. Another important step is updating building codes to require the installation of charging points or wiring for electric vehicles during construction or significant renovation projects^75^. To address the needs of individuals without access to dedicated residential parking, shared charging stations or communal charging facilities in multi-unit residential buildings can be implemented, with corresponding regulations to govern their usage^76^.

### Financial incentives

Both PVs and EVs can come with significant adoption costs. Our findings indicate that high income is a strong predictor of exclusive EV adoption, while those who opt for PVs prioritize potential bill savings. Therefore, providing financial incentives to cover the upfront costs of PVs and EVs jointly is crucial to incentivise co-adoption inclusively. While separate incentives for PV and EV adoption already exist, a more effective approach is to offer bundled incentives that promote the joint adoption of PVs and EVs. This bundling strategy has proven to be successful, as evidenced by a study conducted by Priessner and Hampl^77^, which showed a higher preference for EV purchase when bundled with PV and battery storage. Additionally, the study found that purchase intention for PV was twice as high when offered as a bundle with EV, compared to being considered as a standalone option. By offering bundled incentives, households can benefit from the synergistic advantages of adopting both PVs and EVs (such as bi-directional charging), leveraging the advantages of both technologies. It is worth noting that this bundling option is not limited to current owners of detached houses; it can also take the form of EV and shared solar (community solar), which has been found to significantly increase the willingness to adopt these technologies^78^.

### Increasing technology awareness and interest

The aforementioned policy recommendations primarily tackle the limitations posed by living situations and financial factors. However, there may still be individuals who choose not to adopt PVs and EVs, even in the absence of these constraints. This decision is predominantly influenced by their lack of awareness, interest and/or acceptance of these technologies. Our study reveals the substantial impact of technological interest for all three adoption groups, especially the co-adopters. Therefore, policies should prioritise increasing the general population's interest in these technologies to promote joint adoption. In line with Rogers' adoption of innovations process^79^, we develop the following advice:

1. Increasing knowledge and addressing limited consumer awareness about EVs and PVs seems crucial for adoption, as the majority of people in our sample fall under the group of non-adopters. Reduced interest in technology is particularly prevalent among specific segments of society such as older individuals, those with lower income, and females (as shown in Table 4). To tackle this challenge, targeted outreach programs should be designed for these segments. Campaigns can motivate individuals to learn about the benefits of PV and EV technologies by offering incentives and rewards for participation.
2. To effectively promote the joint adoption of PV and EV technologies, consumers need to perceive substantial advantage and compatibility between the technologies and their needs. They also need to find it easy and simple to leverage PV generation to charge EVs. In this sense, campaigns need to emphasise the numerous positive benefits (financial, environmental, social, and grid-related) associated with using these technologies while educating about the charging practice, such as duration and installation requirements to decrease the perceived complexity of joint adoption. Specific co-adoption strategies can target groups already interested in only one technology.
  i. Our research shows existing or potential PV adopters often live in detached houses, have high electricity consumption (large households/appliance use), or are retirees. In this sense, they are either interested in saving on energy bills or achieving energy security and self-sufficiency^22,27,30^. To encourage these groups to also adopt EVs, campaigns should highlight cost efficiency of EVs when paired with existing PV systems and the potential for bi-directional charging to leverage self-sufficiency. Emphasising the benefits of free residential charging and their flexibility during peak solar hours can effectively target retirees. Given their potentially lower interest in technology, it is crucial to demonstrate the easy compatibility of EVs with PVs and existing systems.
  ii. Technology interest and affordability are less of a barrier for existing or potential EV adopters. Campaigns could focus on the technological advancements of PV and EV integration (smart home management, bi-directional charging). However, living arrangements in denser urban settings might be a constraint. Innovative business models for PV installation in multiunit buildings may be necessary. Highlight environmental benefits and compatibility of solar charging with their telecommuting lifestyles (parked cars during peak solar generation) to resonate with this group.
3. Enabling trialability and observability is crucial to provide tangible experiences and showcase the benefits of co-adoption. Implementing initiatives such as pilot programs (trials) or demonstrations can be highly effective in this regard, as shown by previous research on PV and EV separate adoption^80^. Pilots may be more feasible for existing technology users, while demonstrations offer non-adopters a first-hand experience.

## Conclusion

This study utilised an ICLV model to identify key sociodemographic and attitudinal factors that influence the joint or separate adoption of PV and EV technologies in Australia. The findings identify important differences between consumer segments that are likely to be non-adopters, only adopt PV or EV, or adopt both. Attitudes are among the variables that show greater distinction between consumer groups. While both environmental and technology attitudes play a significant role in shaping adoption patterns, technology interest is by far the most influential attitudinal predictor for co-adoption. PV adoption (alone or together with EVs), however, is also highly dependent on living arrangements, which indicates that co-adoption will only become a widespread reality if regulations and novel business/service models make PV installations a feasible solution to multiunit buildings and rental properties.

To maximise the environmental benefits of PVs and EVs, policies promoting their co-adoption are crucial. While both technologies offer individual advantages (clean electricity from PVs and reduced tailpipe emissions from EVs), their combined impact is far greater. Co-adoption unlocks a powerful synergy: excess solar energy generated by rooftop PV systems can directly charge EVs, maximising renewable energy use for electricity and transportation. This reduces reliance on fossil fuels and associated greenhouse gas emissions across both sectors. Furthermore, bi-directional charging in some EVs allows them to feed surplus solar energy back into the grid during peak periods, promoting grid stability and maximising self-consumption.

Our findings inform policy recommendations focused on co-adoption due to its significant sustainability potential. A one-size-fits-all policy approach is not suitable for promoting the joint adoption of PV and EV, as each adoption group has unique characteristics and faces different barriers. Therefore, policy interventions should target specific needs in three key areas: (a) reducing dependence on living situations for PV and EV adoption, (b) providing bundled financial incentives, and (c) increasing technology interest, as discussed in detail in section "Policy implications". For instance, those currently interested in EV ownership only usually have high income and a strong interest in technology. Co-adoption campaigns targeting this group should thus highlight the potential for experimenting with groundbreaking PV-EV integration technology, while ensuring the feasibility of PV installation in denser urban settings (multi-unit buildings). On the other hand, those currently interested in exclusive PV ownership may be more responsive to the cost-saving opportunity brought by co-adoption. Therefore, interventions targeting this group should show tangible numbers that testify to the cost efficiency of co-adoption. Among non-adopters, it is crucial to enhance awareness and technology familiarity, particularly among female household heads and older individuals.

Despite the insightful results, this study faced some limitations that can be further investigated in future work. Firstly, the sample used in this study was from Australia, a country that lags behind other developed economies in EV adoption. As a result, the number of EV owners in the sample was limited, and current and prospective EV owners were grouped together. Future research should differentiate between current and prospective technology owners (both PV and EV). Secondly, while the study examined the impact of sociodemographic variables and attitudes towards technology and the environment on PV and EV adoption, other factors such as personal values and beliefs as well as specific technology attributes and costs are very important. Future research should consider including social and cultural factors (including social norms and peer influence), and alternative specific attributes like purchase/installation cost, installation capacity for PVs, and vehicle attributes for EVs. Finally, the sample utilised in this study was primarily gathered from an energy-related perspective. Future research should expand its scope to encompass transportation-related variables, thus enabling a more comprehensive analysis. This approach will aid in the development of policy recommendations that account for travel behaviour and prevent incentivising unnecessary vehicle usage.

## Data Availability

The data used in the current study is publicly available from Energy Consumer Australia. Available at: https://ecss.energyconsumersaustralia.com.au/wp-content/uploads/2022/11/ECBS-W13a-Oct22-Data-Pack.zip.

## References

[1] Hardman S (2018). A review of consumer preferences of and interactions with electric vehicle charging infrastructure. Transp. Res. Part D: Transport Environ..

[2] Lavieri P, Domenech C (2021). Electric Vehicle Uptake and Charging: A Consumer-Focused Review.

[3] Lavieri P, Oliveira G (2023). Planning for the Majorities: Are the Charging Needs and Preferences of Electric Vehicle Early Adopters Similar to those of Mainstream Consumers.

[4] Hajhashemi, E., Lavieri, P. S. & Nassir, N.. *Identifying Electric Vehicle Charging Styles Among Consumers: A Latent Class Cluster Analysis* (2023). Available from: https://ssrn.com/abstract=4433893.

[5] Hoarau Q, Perez Y (2018). Interactions between electric mobility and photovoltaic generation: A review. Renew. Sustain. Energy Rev..

[6] Liang J, Qiu Y, Xing B (2022). Impacts of the co-adoption of electric vehicles and solar panel systems: Empirical evidence of changes in electricity demand and consumer behaviors from household smart meter data. Energy Econ..

[7] Australian PV Institute. *Mapping Australian Photovoltaic installations*. 2021 03/04/2023]; Available from: https://pv-map.apvi.org.au/historical.

[8] Kobashi T (2020). Techno-economic assessment of photovoltaics plus electric vehicles towards household-sector decarbonization in Kyoto and Shenzhen by the year 2030. J. Clean. Prod..

[9] Kobashi T (2020). On the potential of “Photovoltaics + Electric vehicles” for deep decarbonization of Kyoto’s power systems: Techno-economic-social considerations. Appl. Energy.

[10] Martin H (2022). Using rooftop photovoltaic generation to cover individual electric vehicle demand—A detailed case study. Renew. Sustain. Energy Rev..

[11] Abou-Zeid, M. & Ben-Akiva, M. Hybrid choice models, In *Handbook of choice modelling,* 383–412 (Edward Elgar Publishing, 2014).

[12] Ausgrid. *NSW Electric Vehicle Owners Survey: Summary Report*. 2020 30/04/2023]; Available from: https://www.ausgrid.com.au/-/media/Documents/Demand-Mgmt/DMIA-research/EV-Owners-Survey-Summary-Report.pdf?la=en&hash=0C623383DFD414A006C05C1FE685D1C1B213FAD3.

[13] Fevang E (2021). Who goes electric? The anatomy of electric car ownership in Norway. Transp. Res. Part D Transport Environ..

[14] Haustein S, Jensen AF (2018). Factors of electric vehicle adoption: A comparison of conventional and electric car users based on an extended theory of planned behavior. Int. J. Sustain. Transp..

[15] Hardman S, Shiu E, Steinberger-Wilckens R (2016). Comparing high-end and low-end early adopters of battery electric vehicles. Transp. Res. Part A Policy Pract..

[16] Brückmann G, Willibald F, Blanco V (2021). Battery Electric Vehicle adoption in regions without strong policies. Transp. Res. Part D Transport Environ..

[17] Sovacool BK (2018). The demographics of decarbonizing transport: The influence of gender, education, occupation, age, and household size on electric mobility preferences in the Nordic region. Glob. Environ. Change.

[18] Lee JH, Hardman SJ, Tal G (2019). Who is buying electric vehicles in California? Characterising early adopter heterogeneity and forecasting market diffusion. Energy Res. Soc. Sci..

[19] Lane BW (2018). All plug-in electric vehicles are not the same: Predictors of preference for a plug-in hybrid versus a battery-electric vehicle. Transp. Res. Part D: Transp. Environ..

[20] Noppers EH (2015). The adoption of sustainable innovations: The role of instrumental, environmental, and symbolic attributes for earlier and later adopters. J. Environ. Psychol..

[21] Peters AM, van der Werff E, Steg L (2018). Beyond purchasing: Electric vehicle adoption motivation and consistent sustainable energy behaviour in The Netherlands. Energy Res. Soc. Sci..

[22] Hansen AR, Jacobsen MH, Gram-Hanssen K (2022). Characterizing the Danish energy prosumer: Who buys solar PV systems and why do they buy them?. Ecol. Econ..

[23] Sommerfeld J (2017). Influence of demographic variables on uptake of domestic solar photovoltaic technology. Renew. Sustain. Energy Rev..

[24] Briguglio M, Formosa G (2017). When households go solar: Determinants of uptake of a Photovoltaic Scheme and policy insights. Energy Policy.

[25] Ameli N, Brandt N (2015). Determinants of households’ investment in energy efficiency and renewables: Evidence from the OECD survey on household environmental behaviour and attitudes. Environ. Res. Lett..

[26] Best R, Burke PJ, Nishitateno S (2019). Understanding the determinants of rooftop solar installation: evidence from household surveys in Australia. Aust. J. Agric. Resour. Econ..

[27] Zander KK (2020). Unrealised opportunities for residential solar panels in Australia. Energy Policy.

[28] McCabe A, Pojani D, Broese van Groenou A (2018). Social housing and renewable energy: Community energy in a supporting role. Energy Res. Soc. Sci..

[29] Cohen J (2019). Q-complementarity in household adoption of photovoltaics and electricity-intensive goods: The case of electric vehicles. Energy Econ..

[30] Bondio S, Shahnazari M, McHugh A (2018). The technology of the middle class: Understanding the fulfilment of adoption intentions in Queensland's rapid uptake residential solar photovoltaics market. Renew. Sustain. Energy Rev..

[31] Zander KK (2019). Preferences for and potential impacts of financial incentives to install residential rooftop solar photovoltaic systems in Australia. J. Clean. Prod..

[32] Lan H (2020). An evaluation of feed-in tariffs for promoting household solar energy adoption in Southeast Queensland, Australia. Sustain. Cities Soc..

[33] Schelly C (2014). Residential solar electricity adoption: What motivates, and what matters? A case study of early adopters. Energy Res. Soc. Sci..

[34] Rai V, Reeves DC, Margolis R (2016). Overcoming barriers and uncertainties in the adoption of residential solar PV. Renew. Energy.

[35] Delmas MA, Kahn ME, Locke SL (2017). The private and social consequences of purchasing an electric vehicle and solar panels: Evidence from California. Res. Econ..

[36] Gezelius M, Mortazavi R (2022). Effect of having solar panels on the probability of owning battery electric vehicle. World Electr. Veh. J..

[37] Gu G (2019). Influence of the adoption of new mobility tools on investments in home renewable energy equipment: Results of a stated choice experiment. Sustain. Cities Soc..

[38] Sharda S (2024). The electric vehicles-solar photovoltaics Nexus: Driving cross-sectoral adoption of sustainable technologies. Renew. Sustain. Energy Rev..

[39] Daly A (2012). Using ordered attitudinal indicators in a latent variable choice model: A study of the impact of security on rail travel behaviour. Transportation.

[40] Hess S (2018). Analysis of mode choice for intercity travel: Application of a hybrid choice model to two distinct US corridors. Transp. Res. Part A Policy Pract..

[41] Hess S, Palma D (2019). Apollo: A flexible, powerful and customisable freeware package for choice model estimation and application. J. Choice Modell..

[42] Energy Consumers Australia. *Energy Consumer Behaviour Survey*. 2022 10/21/2022]; Available from: https://ecss.energyconsumersaustralia.com.au/downloads/

[43] Australian Bureau of Statistics. *National, state and territory population, Dec 2020*. 2021 16/08/2021]; Available from: https://www.abs.gov.au/statistics/people/population/national-state-and-territory-population/dec-2020.

[44] Australian Bureau of Statistics. *Survey of Income and Housing 2017–18*. 2019 16/08/2021]; Available from: https://www.abs.gov.au/statistics/detailed-methodology-information/concepts-sources-methods/survey-income-and-housing-user-guide-australia/latest-release.

[45] Australian Bureau of Statistics. *Housing census, 2021*. 2021 03/04/2023]; Available from: https://www.abs.gov.au/statistics/people/housing/housing-census/latest-release.

[46] Victorian Auditor-General. *Realising the Benefits of Smart Meters*. 2015 08/07/2023]; Available from: https://www.audit.vic.gov.au/sites/default/files/20150916-Smart-Meters.pdf.

[47] McCright AM (2010). The effects of gender on climate change knowledge and concern in the American public. Popul. Environ..

[48] Milfont TL, Sibley CG (2016). Empathic and social dominance orientations help explain gender differences in environmentalism: A one-year Bayesian mediation analysis. Pers. Individ. Differ..

[49] Liu X, Vedlitz A, Shi L (2014). Examining the determinants of public environmental concern: Evidence from national public surveys. Environ. Sci. Policy.

[50] Gilg A, Barr S, Ford N (2005). Green consumption or sustainable lifestyles? Identifying the sustainable consumer. Futures.

[51] Cai Z, Fan X, Du J (2017). Gender and attitudes toward technology use: A meta-analysis. Comput. Educ..

[52] Ardies J (2015). Students attitudes towards technology. Int. J. Technol. Design Educ..

[53] Royne MB, Levy M, Martinez J (2011). The public health implications of consumers' environmental concern and their willingness to pay for an eco-friendly product. J. Consum. Aff..

[54] Rhead R, Elliot M, Upham P (2018). Using latent class analysis to produce a typology of environmental concern in the UK. Soc. Sci. Res..

[55] Twenge JM (2013). Does online social media lead to social connection or social disconnection?. J. Coll. Character.

[56] Helsper EJ, Eynon R (2010). Digital natives: Where is the evidence?. Br. Educ. Res. J..

[57] Fukuoka Y (2019). Short-and long-term effects of a mobile phone app in conjunction with brief in-person counseling on physical activity among physically inactive women: The mPED randomized clinical trial. JAMA Netw. Open.

[58] Shen J, Saijo T (2008). Reexamining the relations between socio-demographic characteristics and individual environmental concern: Evidence from Shanghai data. J. Environ. Psychol..

[59] Inglehart R (1995). Public support for environmental protection: Objective problems and subjective values in 43 societies. PS Polit. Sci. Polit..

[60] Liu N, Yu R (2017). Identifying design feature factors critical to acceptance and usage behavior of smartphones. Comput. Hum. Behav..

[61] Lavieri PS (2017). Modeling individual preferences for ownership and sharing of autonomous vehicle technologies. Transp. Res. Rec..

[62] Bhat CR (2015). A comprehensive dwelling unit choice model accommodating psychological constructs within a search strategy for consideration set formation. Transp. Res. Part B Methodol..

[63] Kang S (2021). Pooled versus private ride-hailing: A joint revealed and stated preference analysis recognizing psycho-social factors. Transp. Res. Part C Emerg. Technol..

[64] Aguilera-García Á (2022). Behavioral factors impacting adoption and frequency of use of carsharing: A tale of two European cities. Transp. Policy.

[65] Esteves J, Alonso-Martínez D, de Haro G (2021). Profiling spanish prospective buyers of electric vehicles based on demographics. Sustainability.

[66] Daniel TO, Stanton CM, Epstein LH (2013). The future is now: Reducing impulsivity and energy intake using episodic future thinking. Psychol. Sci..

[67] Frederiks ER, Stenner K, Hobman EV (2015). Household energy use: Applying behavioural economics to understand consumer decision-making and behaviour. Renew. Sustain. Energy Rev..

[68] Brakels, R. ACT Pensioner Rebates For Solar & To Help Ditch Gas: How To Apply. 2023 03/04/2023]; Available from: https://www.solarquotes.com.au/blog/act-pensioner-rebates-solar/#:~:text=How%20To%20Apply-,ACT%20Pensioner%20Rebates%20For%20Solar%20%26%20To,Ditch%20Gas%3A%20How%20To%20Apply&text=If%20you're%20an%20ACT,rebate%20of%20up%20to%20%242%2C500.

[69] Electric Vehicle Council. *State of Electric Vehicles* 2022 09/07/2023 04/05/2023]; Available from: https://electricvehiclecouncil.com.au/wp-content/uploads/2022/03/EVC-State-of-EVs-2022.pdf.

[70] Aman S, Simmhan Y, Prasanna VK (2013). Energy management systems: state of the art and emerging trends. IEEE Commun. Mag..

[71] Lavieri PS, Bhat CR (2019). Investigating objective and subjective factors influencing the adoption, frequency, and characteristics of ride-hailing trips. Transp. Res. Part C Emerg. Technol..

[72] Solar Victoria. *Solar rebates for rental properties*. 2019 02/03/2023]; Available from: https://www.solar.vic.gov.au/solar-rebates-rental-properties.

[73] Horváth D, Szabó RZ (2018). Evolution of photovoltaic business models: Overcoming the main barriers of distributed energy deployment. Renew. Sustain. Energy Rev..

[74] Reis IFG (2022). Collective self-consumption in multi-tenancy buildings–To what extent do consumers’ goals influence the energy system's performance?. Sustain. Cities Soc..

[75] Hall D, Lutsey N (2020). Electric Vehicle Charging Guide for Cities.

[76] Hall D, Lutsey N (2017). Emerging Best Practices for Electric Vehicle Charging Infrastructure.

[77] Priessner A, Hampl N (2020). Can product bundling increase the joint adoption of electric vehicles, solar panels and battery storage? Explorative evidence from a choice-based conjoint study in Austria. Ecol. Econ..

[78] Stauch A (2021). Does solar power add value to electric vehicles? An investigation of car-buyers’ willingness to buy product-bundles in Germany. Energy Res. Soc. Sci..

[79] Rogers EM (2003). Diffusion of Innovations.

[80] Gerdesics V (2013). Diffusion of renewable energy innovations—Innovation-acceptance behaviour of the Hungarian society. Közgazd. Fórum.

